# Interplay between the abdominopelvic radiotherapy and gut microbiota

**DOI:** 10.3389/fmicb.2025.1692179

**Published:** 2025-12-12

**Authors:** Ding Chenxiang, Zhuang Jing, Yu Xiaojian, Xie Hua, Yang Xi

**Affiliations:** 1 Huzhou Central Hospital, Fifth School of Clinical Medicine of Zhejiang Chinese Medical University, Huzhou, China; 2 Huzhou Central Hospital, Affiliated Central Hospital Huzhou University, Huzhou, China; 3 Bengbu Medical University Graduate School, Bengbu, China; 4 Xuancheng People’s Hospital, Affiliated Xuancheng Hospital of Wannan Medical College, Xuancheng, China; 5 Zhejiang-France United Laboratory of Integrated Traditional Chinese and Modern Medicine in Colorectal Cancer, Huzhou, China

**Keywords:** gut microbiota, radiotherapy, abdominopelvic malignant tumor, radiation-induced intestinal injury, radiosensitization

## Abstract

Radiotherapy is a crucial treatment modality for abdominopelvic malignancies, with particularly significant effects on the gut microbiota. A reciprocal relationship exists between abdominopelvic radiotherapy and the gut microbiota. The gut microbiota plays vital roles in maintaining host health, modulating immune responses, and regulating metabolic functions. Abdominopelvic radiotherapy induces significant alterations in both the diversity and abundance of the gut microbiota, which may be critically involved in the development of radiotherapy-related adverse effects and the compromised therapeutic efficacy. Concurrently, the distinct biological properties of the gut microbiota and its derivatives can also influence the host response to radiotherapy. Understanding this interplay between abdominopelvic radiotherapy and the gut microbiota is of paramount importance for improving patient quality of life and treatment outcomes. This review discusses the impact of abdominopelvic radiotherapy on gut microbiota composition and summarizes recent advances in microbiota-targeted interventions aimed at radioprotection and radiosensitization, providing a theoretical foundation for optimizing radiotherapy for abdominopelvic malignancies.

## Graphical abstract

**Figure:**
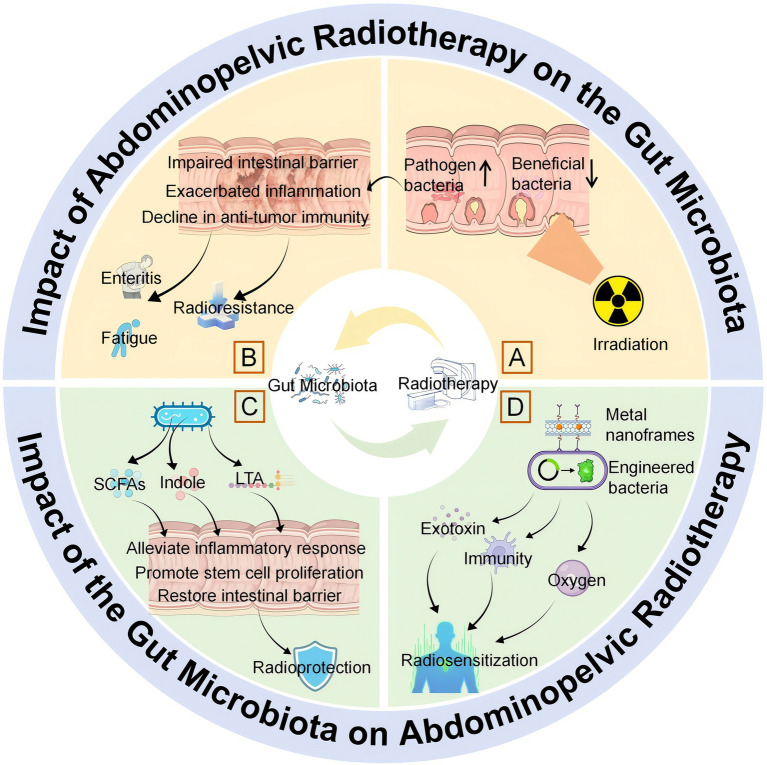
Interplay between the abdominopelvic radiotherapy and gut microbiota. On one hand, irradiation of the abdominopelvic region inevitably induces intestinal injury and gut microbiota dysbiosis, characterized by a reduction in beneficial bacteria and an expansion of potentially pathogenic species (A). This microbial dysbiosis compromises intestinal barrier integrity, exacerbates local and systemic inflammation, and impairs anti-tumor immunity, thereby contributing to adverse effects such as radiation enteritis and fatigue, and potentially diminishing tumor radiosensitivity (B). Conversely, gut microbiota-derived metabolites, including SCFAs and indole derivatives, as well as bacterial structural components (e.g., LTA), can activate specific signaling pathways to promote epithelial repair, enhance barrier function, and suppress inflammatory responses, thereby mediating radioprotective effects (C). Furthermore, leveraging the anaerobic tropism and immunomodulatory capacity of certain bacteria, combined with synthetic biology and nanotechnology, enables the construction of engineered strains or biohybrid systems for targeted toxin delivery, amelioration of tumor hypoxia, and remodeling of the immunosuppressive microenvironment, which offers novel strategies for radiosensitization (D).

## Highlights

- An interplay exists between abdominopelvic radiotherapy and the gut microbiota.
- Abdominopelvic radiotherapy reduces the α-diversity of the gut microbiota, decreases the abundance of beneficial microbial populations, and increases the abundance of harmful ones.
- Gut microbiota dysbiosis contributes to radiation-induced enteritis and fatigue following abdominopelvic radiotherapy and compromises therapeutic efficacy.
- Gut microbiota-derived metabolites mediate intestinal radioprotection through multiple mechanisms after abdominopelvic radiotherapy.
- The integration of gut microbiota with bioengineering techniques represents a promising future direction for achieving radiosensitization.

## 1 Introduction

Abdominopelvic malignancies are lesions that occur in the abdomen and pelvis, originating from various organs and tissues in this region. According to the latest cancer statistics published in 2024, gynecological cancers, colorectal cancer, and prostate cancer rank highest in both incidence and mortality among abdominopelvic malignancies (Bray et al., 2024). Currently, surgery, chemotherapy, radiotherapy, immunotherapy, targeted therapy, and endocrine therapy are the main treatment modalities for abdominopelvic malignancies (Abu-Rustum et al., 2023). Radiotherapy exerts its antitumor effects primarily by utilizing high-energy radiation to directly damage the DNA structure of cancer cells. Additionally, radiation interacts with intracellular water molecules to generate free radicals, inducing extensive DNA damage (Nikitaki et al., 2022). It is particularly indicated for patients ineligible for surgery, as postoperative adjuvant treatment, or for recurrent cases (Tubridy et al., 2024; Bahadoer et al., 2021; Hasan et al., 2016), holding a significant position in the comprehensive management of abdominopelvic malignancies. Notably, radiotherapy directed at the abdominopelvic region, which includes the gastrointestinal tract, can significantly disrupt gut microbiota homeostasis, leading to dysbiosis (Moraitis et al., 2023). This dysbiosis not only contributes to the development of radiotherapy-related side effects but also diminishes the radiosensitivity of tumor tissues (Lu et al., 2024; Li et al., 2022).

Over 97% of human commensal bacteria reside within the digestive tract, predominantly in the colon, collectively forming the gut microbiota (Sender et al., 2016). Kåhrström et al. (2016) demonstrated that 98% of the gut microbiota belongs to four dominant phyla: *Bacteroidetes*, *Firmicutes*, *Proteobacteria*, and *Actinobacteria*. These commensal microorganisms play crucial roles in essential physiological processes, including regulating intestinal epithelial cell proliferation and differentiation, activating innate immune responses, and participating in metabolic homeostasis regulation (Belkaid and Hand, 2014). They can also modulate the efficacy of various cancer therapies, including radiotherapy (Helmink et al., 2019; Gately, 2019). Gut microbiota-derived short-chain fatty acids (SCFAs) promote the production of tight junction proteins and mucins, thereby maintaining intestinal barrier integrity following radiotherapy (He et al., 2023). Outer membrane vesicles (OMVs) secreted by the gut microbiota contain various bacterial-associated molecular patterns and serve as potent mediators for enhancing anti-tumor immunity (Huang X. et al., 2021). Furthermore, the anaerobic or facultative anaerobic characteristics of gut microbiota enable their effective targeting of hypoxic tumor regions. Combined with bioengineering technologies, they can ameliorate hypoxia within the tumor microenvironment (TME), consequently enhancing radiosensitivity. In summary, a close interaction exists between the gut microbiota and radiotherapy. Understanding the mechanisms underlying the interplay between abdominopelvic radiotherapy and the gut microbiota will provide a theoretical foundation for developing microbiota-modulating adjuvant strategies for radiotherapy. This approach aims to achieve the dual goals of mitigating adverse effects and enhancing anti-tumor efficacy.

## 2 Impact of abdominopelvic radiotherapy on the gut microbiota

### 2.1 Abdominopelvic radiotherapy alters the diversity and abundance of the gut microbiota

The gut microbiota is influenced by numerous factors, among which abdominopelvic radiotherapy is one that significantly alters its diversity and abundance. A systematic review incorporating 11 studies demonstrated that exposure to radiation induces changes in intestinal bacterial composition, characterized by a reduction in beneficial bacteria and an increase in potentially harmful bacteria (Fernandes et al., 2021). Due to such alterations, the gut microbiota may serve as a novel biomarker for radiation exposure (Lam et al., 2012).

In animal models, Zhao et al. (2020) confirmed that abdominal irradiation disrupts gut microbiota balance in mice and significantly reduces the alpha-diversity index. Following radiation exposure, the abundance of the *Bacteroidetes*, *Firmicutes*, and *Actinobacteria* decreased in the gut, while the abundance of *Proteobacteria*, which is enriched with pathogenic bacteria, significantly increased. Duan et al. (2022) further revealed a pathogen-probiotic dynamic imbalance in the gut microbiota of irradiated mice: the abundance of opportunistic pathogens such as *Escherichia* and *Shigella* (*Proteobacteria*) increased, whereas the abundance of beneficial bacteria including *Romboutsia* (*Firmicutes*) and *Bacteroides* (*Bacteroidetes*) decreased. Lu et al. (2025) also found that abdominal irradiation induces a “pathogenic remodeling” of the intestinal microenvironment, leading to *Enterobacteriaceae* becoming the dominant bacterial family. Collectively, these findings indicate that abdominal radiation exposure induces gut microbial dysbiosis in animal models.

Clinically, alterations in the gut microbiota following abdominopelvic radiotherapy exhibit consistent trends among patients, irrespective of the presence of radiation-induced intestinal injury. He et al. (2023) observed that, compared to cervical cancer patients who did not receive radiotherapy, patients undergoing radiotherapy exhibited a significant reduction in fecal microbiota alpha-diversity, increased abundance of *Proteobacteria*, and decreased abundance of *Firmicutes* and *Bacteroidetes*. Nam et al. (2013) compared fecal microbiota changes before and after pelvic radiotherapy in nine gynecological cancer patients, reporting a 10% decrease in *Firmicutes* and a 3% increase in *Fusobacterium*. *Bacteroidetes*, which typically confer beneficial functions within the gut (Wexler, 2007), exhibit variable abundance changes across studies. While most studies observed a decrease in its abundance (He et al., 2023; Yi et al., 2021; Wang et al., 2019), a minority reported an increase (Sahly et al., 2019). Furthermore, in patients developing radiation-induced intestinal injury post-radiotherapy, some studies also observed an elevated abundance of *Actinobacteria* (Manichanh et al., 2008). Collectively, these findings reveal characteristic alterations associated with radiotherapy-induced gut microbiota dysbiosis: diminished alpha-diversity accompanied by an imbalance in the ratio of pathogenic to beneficial bacteria.

In summary, current research indicates that radiotherapy reduces the alpha-diversity and alters the abundance of the gut microbiota in patients with abdominopelvic malignancies (Table 1). The abundance of the *Firmicutes* and *Bacteroidetes* decreases. These phyla predominantly comprise probiotic bacteria whose exposed peptidoglycan structures are highly susceptible to degradation by radiation-generated reactive oxygen species (ROS) (Roy and Trinchieri, 2017). Conversely, the abundance of the *Proteobacteria* and *Actinobacteria* increases. These phyla encompass various opportunistic pathogens, exemplified by *Escherichia coli*, which can activate the RecA-mediated SOS DNA damage response system (Kaushik et al., 2022). This activation enhances their capacity for repairing radiation-induced double-strand breaks, thereby conferring a competitive advantage within the intestinal microenvironment. The subsequent overproliferation of these harmful bacteria further suppresses the colonization and growth of beneficial bacterial populations, ultimately establishing a vicious cycle.

**Table 1:** Radiotherapy affects diversity and abundance of gut microbiota.

References	Samples	Radiotherapy setting	Disease	Alterations in the gut microbiota composition
Zhao et al. (2020)	Feces of male C57BL/6 J mice Radiotherapy (*n* = 3) Non-radiotherapy (*n* = 4)	High-dose abdominal precision radiation with a single dose of 10 Gy	–	*Proteobacteria↑* *Bacteroidete↓* *Firmicutes↓* *Actinobacteria↓*
Duan et al. (2022)	Feces of male SPF-grade Wistar rats Radiotherapy (*n* = 8); Non-radiotherapy (*n* = 8)	Irradiation with a 6-MV X-ray beam delivering 22 Gy at a dose rate of 300 cGy/min	–	*Proteobacteria↑* *Shigella↑* *Escherichia↑* *Bacteroidete↓* *Firmicutes↓* *Lactobacillus↓* *Ruminococcus↓*
Lu et al. (2025)	Feces of male C57BL/6 J mice Radiotherapy (*n* = 10); Non-radiotherapy (*n* = 10)	A single dose of 13 Gy X ray at a rate of 1.0 Gy/min	–	*Proteobacteria↑* *Enterobacteriaceae↑*
Manichanh et al. (2008)	Pre-post-radiotherapy fecal samples from patients (*n* = 5)	Abdominal radiotherapy	Abdominal tumor	*Actinobacteria↑* *Bacilli↑* *Clostridia↓*
Nam et al. (2013)	Pre-post-radiotherapy fecal samples from patients (*n* = 9)	Pelvic radiotherapy	Gynecologic cancer	*Fusobacteria↑* *Clostridiaceae↑* *Streptococcaceae↑* *Fusobacteriaceae↑* *Firmicutes↓* *Bacteroidetes↓* *Eubacteriaceae↓*
Wang et al. (2019)	Pre-/post-radiotherapy fecal samples from patients (*n* = 18)	Pelvic radiotherapy	Cervical cancer	*Proteobacteria↑* *Gammaproteobacteria↑* *Enterobacteriaceae↑* *Phyllobacteriaceae↑* *Bacteroidetes↓* *Ruminococcaceae↓*
Sahly et al. (2019)	Pre-/post-radiotherapy fecal samples from pediatric patients (*n* = 3)	Pelvic radiotherapy	Rhabdomyosarcoma	*Proteobacteria↑* *Bacteroidete↑* *Actinobacteria↑* *Firmicutes↓*
He et al. (2023)	Feces of patients Radiotherapy (*n* = 137); Non-radiotherapy (*n* = 128)	Pelvic radiotherapy	Cervical cancer	*Proteobacteria↑* *Romboutsia↑* *Escherichia-Shigella↑* *Bacteroidete↓* *Firmicutes↓* *A. muciniphila↓* *Lactobacillus↓* *Collinsella↓* *Bacteroides↓* *Blautia↓*

The meaning of a symbol in the table: ↑, increased; ↓, decreased.

### 2.2 Gut microbiota dysbiosis induces adverse effects in abdominopelvic radiotherapy

A stable gut microbiota is essential for maintaining the intestinal epithelial barrier, suppressing inflammation, and preserving host immune homeostasis. Dysbiosis of the gut microbiota not only compromises intestinal epithelial barrier function but also promotes the expression of inflammatory cytokines and activates systemic inflammatory responses. These pathological changes can lead to the development of various radiotherapy-induced adverse effects, among which radiation enteritis (RE) is the most prevalent.

RE represents the most prevalent adverse effect of abdominopelvic radiotherapy. This pathology is characterized by persistent intestinal tissue damage and extensive pathological alterations, manifesting with complex clinical presentations and a protracted clinical course. RE significantly impairs patients’ quality of life and compromises comprehensive cancer treatment efficacy (Zhu and Li, 2020; Moraitis et al., 2023; Segers et al., 2019). Characteristic symptoms include diarrhea, abdominal pain, constipation, and hematochezia (Hauer-Jensen et al., 2014). Gut microbiota dysbiosis is closely associated with RE development. Wang et al. (2019) analyzed fecal samples from cervical cancer patients undergoing pelvic radiotherapy, identifying distinct dysbiosis signatures in RE patients. Compared to patients without RE, those developing RE exhibited significantly reduced alpha-diversity, increased beta-diversity, elevated relative abundance of *Proteobacteria*, and decreased relative abundance of *Bacteroidetes* and *Firmicutes*. This disparity became more pronounced with increasing RE severity. Bacterial genera such as *Bifidobacterium*, *Bacteroides*, *Lactobacillus*, and *Akkermansia muciniphila* maintain intestinal epithelial barrier integrity by producing SCFAs that stimulate gut cells to secrete tight junction proteins (ZO-1, occludin, and claudin-3) and mucin MUC2 (He et al., 2023; Hsieh et al., 2015; Zhang et al., 2024; La Fata et al., 2018). Furthermore, bacteria including *Faecalibacterium prausnitzii*, *Ruminococcus*, *Coprococcus*, *Dorea*, *Lachnospira*, *Roseburia*, *Bifidobacterium*, and *Clostridium* alleviate intestinal inflammation by suppressing the NF-κB pathway, thereby reducing the expression of inflammatory cytokines such as TNF-α, IL-8, and IL-1β (Sokol et al., 2008; Riedel et al., 2006; Lakhdari et al., 2011). Abdominopelvic radiotherapy substantially reduces the abundance of these barrier-protective and anti-inflammatory bacterial populations. Consequently, intestinal barrier function becomes compromised, permitting overgrowth of harmful bacteria. This leads to accumulation of endotoxins in the bloodstream, elevation of inflammatory cytokines, and ultimately culminates in the pathogenesis of radiation enteritis.

Beyond gastrointestinal symptoms, patients with RE frequently experience concomitant fatigue (Wang et al., 2015). Clinical studies reveal that, compared to patients without fatigue symptoms at the end of radiotherapy, those reporting fatigue exhibit significant enrichment of the genera *Eubacterium*, *Streptococcus*, *Adlercreutzia*, and *Actinomyces* in their gut microbiota (González-Mercado et al., 2021). Furthermore, among fatigued patients, those in the high-fatigue group demonstrate significantly reduced abundance of genera such as *Subdoligranulum*, *Faecalibacterium*, *Bifidobacterium*, and *Agathobacter* compared to the low-fatigue group (Xiao et al., 2021). Notably, these diminished bacterial taxa serve as primary producers of SCFAs—metabolites with well-established anti-inflammatory properties (Koh et al., 2016). Existing evidence indicates that a lower abundance of SCFA-producing bacterial taxa contributes to a pro-inflammatory state associated with post-radiotherapy fatigue (Xiao et al., 2016). Further functional analyses suggest that inflammatory cytokines entering the systemic circulation act upon the central nervous system, thereby activating the gut-brain axis. This mechanism may represent one key pathway underlying the development of radiotherapy-related fatigue (Xiao et al., 2021; González-Mercado et al., 2020).

Therefore, restoring intestinal microbiota balance represents a significant potential approach for alleviating radiotherapy side effects. Currently, oral probiotics and fecal microbiota transplantation are the two main strategies. Novel oral delivery systems for probiotics, such as microfluidic strategies (Yang et al., 2024) and single-cell encapsulation (Han et al., 2024), aim to ensure precise targeting and stable colonization of probiotics. However, their limited range of bacterial species makes it difficult to compensate for complex microecological imbalances. Although fecal microbiota transplantation can deliver a more complete spectrum of microbiota, the heterogeneity and unpredictability of its efficacy constrain its broad clinical application (Yadegar et al., 2024). Integrating multi-omics data for precise donor-recipient matching and focusing on the restoration of core functional microbial communities may be a feasible direction for achieving effective personalized interventions in the future.

### 2.3 Gut microbiota dysbiosis diminishes radiotherapeutic efficacy

Gut microbiota dysbiosis not only contributes to radiotherapy-induced adverse effects but also negatively correlates with therapeutic response to radiation. Xiao et al. demonstrated that melanoma-bearing mice treated with a broad-spectrum antibiotic cocktail exhibited significantly diminished radiotherapeutic efficacy (Shiao et al., 2021). Clinically, head and neck cancer patients receiving broad-spectrum antibiotics show substantially reduced radiation response rates (Nenclares et al., 2020). These findings suggest that antibiotic-mediated gut dysbiosis may underlie this phenomenon. Furthermore, the Sims TT team observed that radiotherapy-induced reduction in gut microbiota alpha-diversity significantly correlates with decreased chemo-radiotherapy response rates and reduced overall survival in cervical cancer patients (Sims et al., 2021). Yang et al. additionally reported that Shengmai turmeric powder enhances radiotherapeutic efficacy by preserving gut microbiota against radiation-induced dysbiosis, indirectly supporting the inverse relationship between microbial imbalance and treatment outcomes (Yang et al., 2019).

An intact gut microbiota enhances anti-tumor immune responses through immunomodulation, thereby indirectly improving radiosensitivity. Paulos et al. demonstrated that whole-body irradiation in mice induces translocation of gut microbes—including *Enterobacter cloacae*, *Escherichia coli*, *Lactobacillus*, and *Bifidobacterium*—to mesenteric lymph nodes, augmenting anti-tumor effects (Paulos et al., 2007). Bacterial components (e.g., lipopolysaccharides, flagellin, DNA) and metabolites from these anaerobic or facultative anaerobic gut microbes serve as pathogen-associated molecular patterns (PAMPs). These PAMPs activate immune cells via Toll-Like Receptors (TLRs), significantly amplifying anti-tumor immunity (Huang X. et al., 2021; Al-Qadami et al., 2019). Consequently, depletion or dysbiosis of the gut microbiota compromises therapeutic responsiveness to radiotherapy. Notably, bacterial depletion may further provoke intestinal fungal overgrowth. Such overgrowth can suppress anti-tumor immunity by activating the Dectin-1 receptor pathway (Shiao et al., 2021), exerting dual inhibitory effects on radiotherapeutic efficacy. Although fecal microbiota transplantation (FMT) or probiotic administration may restore microbial balance and demonstrate potential in mitigating radiotherapy-induced adverse effects (Jian et al., 2021; Garczyk et al., 2022; Van Dingenen et al., 2023), conclusive evidence for their efficacy in improving radiotherapeutic outcomes remains lacking. Thus, harnessing gut microbiota-mediated immunomodulation may constitute a pivotal strategy for enhancing radiotherapeutic efficacy.

In summary, although the gut microbiota shows potential value in alleviating complications associated with radiotherapy, the direct mechanisms by which it influences radiotherapy efficacy have not yet been fully elucidated. There remains a lack of sufficient clinical evidence to systematically clarify the specific role of gut microbiota in radioresistance, which is undoubtedly an area worthy of further exploration in radiation medicine. In addition, the theory that the gut microbiota indirectly affects radiotherapy efficacy by modulating anti-tumor immune responses has been substantiated. Based on this characteristic of the gut microbiota, combining emerging engineered bacteria and nanotechnology in the future may represent one of the key pathways to achieving its clinical translation.

## 3 Impact of the gut microbiota on abdominopelvic radiotherapy

### 3.1 Protective effects of gut microbiota-derived metabolites in abdominopelvic radiotherapy

**Figure 1:**
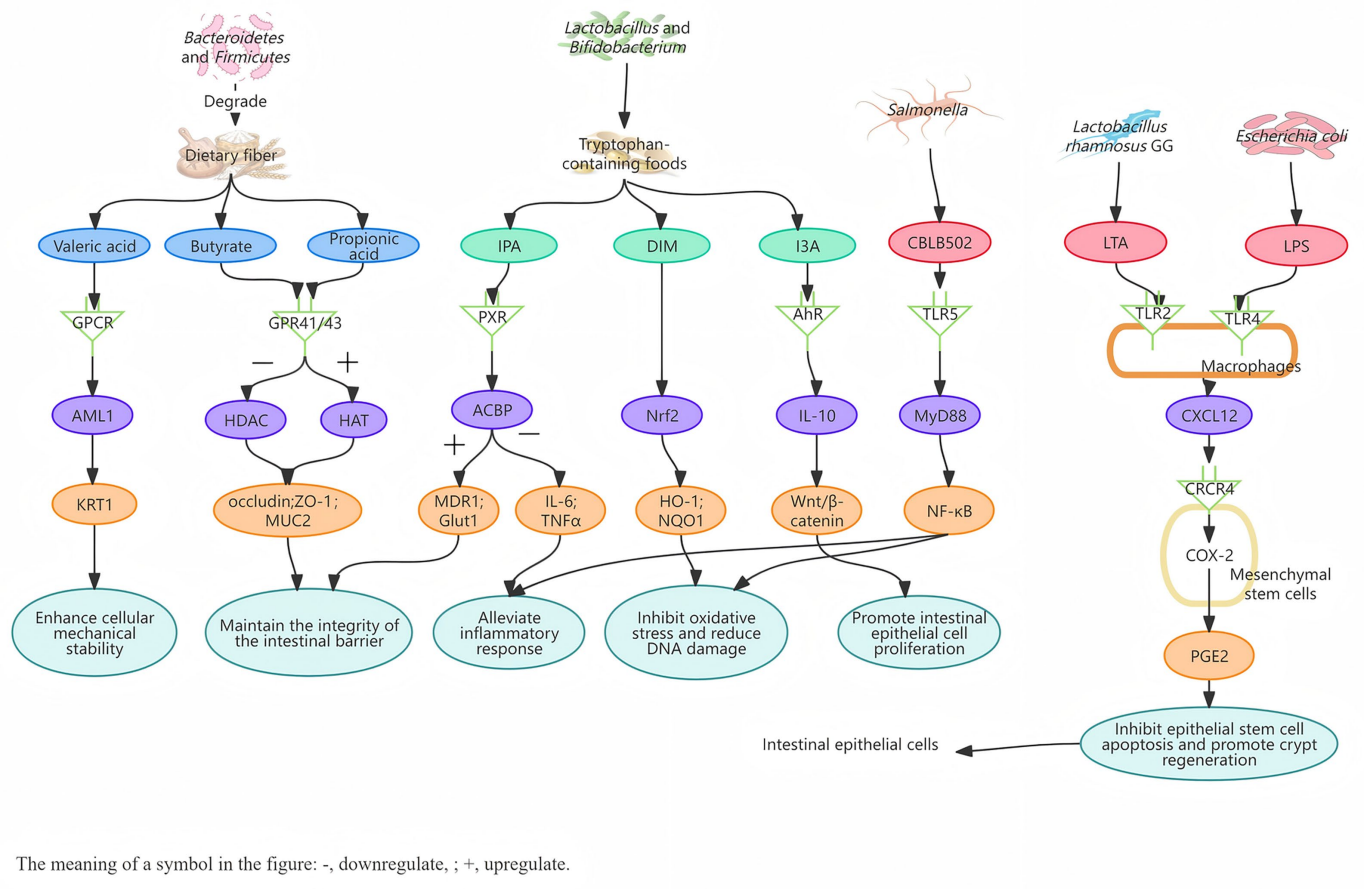
Mechanisms by which gut microbiota derivatives mediate intestinal radioprotection. SCFAs mediate the GPR/HADC signaling pathway, indole metabolites mediate the PXR/ACBP; AhR/IL-1 and Nrf2 pathways, and bacterial cell wall and flagellar components mediate the TLR/PGE2 signaling cascade. Together, they exert multiple radioprotective effects, including inhibiting intestinal inflammation, alleviating oxidative stress, promoting the proliferation of intestinal epithelial cells, and maintaining the integrity of the intestinal barrier.

As the most frequent complication of abdominopelvic radiotherapy, RE has emerged as a critical limiting factor in the advancement of radiation oncology (Hauer-Jensen et al., 2014). Radiotherapy-induced gut microbiota dysbiosis constitutes an underlying mechanism of radiation-associated intestinal injury. Previous studies indicate that oral administration of multi-strain probiotics (Xie et al., 2024b) and FMT (Wang L. et al., 2024; Liu et al., 2023) effectively mitigate radiation-related tissue damage. The mechanistic basis involves synergistic actions through which the gut microbiota alleviates radiation-induced intestinal injury via multiple pathways (Figure 1).

SCFAs and indole metabolites in microbial metabolic products exhibit significant intestinal barrier protective functions. Guo et al. found that “elite survivor” mice, which are tolerant to high doses of radiation, have an enrichment of *Lachnospiraceae* and *Enterococcaceae* in their intestines. The high levels of SCFAs and tryptophan metabolites produced by these microbial groups alleviate hematopoietic and gastrointestinal syndromes and suppress inflammatory responses, thereby providing long-term radioprotection for the mice (Guo et al., 2020). SCFAs are produced by anaerobic microbiota in the gut (such as *Bacteroidetes*, *Firmicutes*, etc.) through the fermentation of dietary fibers (Fusco et al., 2023). They exert their protective roles via specific receptors. Clinical studies have demonstrated that butyrate enemas can significantly alleviate radiation-induced intestinal injury in the abdominopelvic region (Vernia et al., 2000; Hille et al., 2008), possibly via modulation of GPR41/43 receptors on the surface of intestinal epithelial cells (Li et al., 2020b). Propionic acid produced by *Akkermansia muciniphila* binds to the GPR43 receptor, activating histone acetyltransferases (HAT) while inhibiting histone deacetylases (HDAC). This elevates histone acetylation levels, leading to chromatin structure loosening. Consequently, it enhances transcription of tight junction proteins (occludin, ZO-1) and mucin MUC2, ultimately contributing to the maintenance of intestinal epithelial barrier integrity following radiotherapy (He et al., 2023; Zhao et al., 2025). Additionally, valeric acid may activate the AML1/KRT1 signaling pathway through specific transporters or GPCRs, upregulating the expression of cytoskeleton-associated proteins, thereby enhancing intracellular mechanical stability, which could represent one of the mechanisms underlying its radioprotective effects (Li et al., 2020a). The generation of another important class of metabolites—indole derivatives—relies on the tryptophan metabolic enzyme systems of *Lactobacillus*, *Bifidobacterium*, and other gut microbiota (Dodd et al., 2017). Studies indicate that indole-3-propionic acid (IPA) reduces oxidative stress levels via the PXR/ACBP pathway, thereby ameliorating radiation-induced hematopoietic and gastrointestinal toxicity (Wikoff et al., 2009; Xiao et al., 2020). Indole-3-carboxaldehyde (I3A) activates the aryl hydrocarbon receptor (AhR) in intestinal epithelial cells, promoting expression of the anti-inflammatory cytokine IL-10, which facilitates differentiation of intestinal stem cells into functional cells through mediation of the Wnt3/β-catenin signaling pathway, ultimately enhancing intestinal barrier function and repairing radiation-induced damage (Xie et al., 2024a). Furthermore, 3, 3′-diindolylmethane (DIM) contributes to intestinal radioprotection by maintaining the proliferative and differentiative functions of intestinal stem cells, mitigating radiation-associated DNA damage, and activating the Nrf2 pathway to alleviate oxidative stress (Lu et al., 2019). SCFAs and indole metabolites maintain intestinal epithelial repair and reduce both intestinal inflammation and oxidative factor levels following radiotherapy through various mechanisms, thereby exerting radioprotective effects.

Apart from metabolic products, the structural components of the microbiota also possess radioprotective potential (Table 2). The cell wall component lipoteichoic acid (LTA) of *Lactobacillus rhamnosus GG* (LGG) activates the TLR2 receptor on macrophages, triggering the secretion of the chemokine CXCL12, which then binds to the CXCR4 receptor on mesenchymal stem cells (MSCs). This interaction drives their migration from the lamina propria of the small intestinal villi to the surrounding crypts, and by engaging the COX-2/PGE₂ pathway, inhibits apoptosis of epithelial stem cells and promotes crypt regeneration, thereby specifically protecting the intestine from radiation-induced damage (Ciorba et al., 2012; Riehl et al., 2019). Lipopolysaccharides (LPS) from the outer membrane of *Escherichia coli* mediates a similar protective effect through activation of the TLR4 receptor (Lu et al., 2020; Riehl et al., 2000; Stenson and Ciorba, 2020). Furthermore, the derivative CBLB502, obtained from recombinant modified *Salmonella* flagellin, can activate the TLR5 receptor on intestinal epithelial cells, mediating NF-κB signal transduction to induce the production of downstream protective factors (Burdelya et al., 2008). These studies indicate that different structural components of the microbiota, through specific activation of the Toll-Like Receptor (TLR) receptor family, establish differentiated signal transduction networks that ultimately converge on the common biological endpoint of intestinal epithelial protection.

**Table 2:** Mechanisms by which gut microbiota derivatives alleviate radiation-induced intestinal injury.

References	Bacteria	Derivative	Study setting	Type of cancer	Mechanism
Guo et al. (2020)	*Lachnospiraceae; Enterococcaceae*	Propionate; I3A;kynurenic acid(KYNA)	*In vitro*; *in vivo*	–	SCFAs, the tryptophan metabolites KYNA and I3A, provide long-term radioprotection by attenuating DNA damage, reactive oxygen species production, and inflammatory responses
Li et al. (2020b)	*Allobaculum*	Butyrate	*In vivo*	–	Through activation of GPR41/GPR43 receptors, butyrate prevents radiation-induced enteritis by inhibiting inflammatory responses, promoting metabolism and energy recovery, and maintaining intestinal epithelial integrity
He et al. (2023)	*Akkermansia muciniphila*	Propionic acid	*In vivo*	Cervical cancer	Propionic acid promotes the production of tight junction proteins and mucins through the activation of GRP43 receptor and protects the intestinal tract from radiation damage
Zhao et al. (2025)	*Weissella cibaria; Muribaculaceae*	Exopolysaccharides-2 (EPS-2); propionic acid	*In vivo*	–	EPS-2 promotes the production of propionic acid by Muribaculaceae and enhances the function of colonic cuprocytes and mucin content, thereby protecting the intestinal mucosa
Li et al. (2020a)	*–*	Valeric acid	*In vivo*	–	Valeric acid maintains intestinal cell mechanical stability through the AML1/KRT1 signaling axis, while synergistically attenuating radiation-induced intestinal damage by inhibiting inflammatory responses and upregulating tight junction protein expression
Xiao et al. (2020)	*Clostridium*	IPA	*In vivo*	Colorectal cancer; cervical cancer	IPA exerts intestinal protection through the PXR/ACBP signaling pathway
Xie et al. (2024a)	*Lactobacillus*; *Bifidobacterium*	I3A	*In vivo*	Colorectal cancer	I3A protects the gut by upregulating IL-10, activating the Wnt3/β-catenin pathway, promoting stem cell proliferation and differentiation and enhancing tight junction protein expression
Lu et al. (2019)	*–*	DIM	*In vivo*	–	DIM synergistically exerts intestinal radioprotection by maintaining the proliferation and differentiation function of intestinal stem cells, inhibiting radiation-associated DNA damage and activating the Nrf2 pathway to alleviate oxidative stress, among other mechanisms
Riehl et al. (2019)	*Lactobacillus rhamnosus GG*	LTA	*In vitro*; *in vivo*	Colorectal cancer	LTA protects the intestinal epithelium from radiation damage by activating macrophage TLR2 receptors to induce MSCs to migrate to the crypt and express COX-2, which drives PGE2 synthesis
Lu et al. (2020)	*Escherichia-Shigella*	LPS	*In vivo*	–	LPS mediates the intestinal inflammatory response through the TLR4/MyD88 signaling pathway, whereas exogenous PC reduces LPS-producing bacteria, thereby protecting the intestines
Burdelya et al. (2008)	*Salmonella*	CBLB502	*In vitro*; *in vivo*	Sarcoma; melanoma	CBLB502 binds TLR5 receptor to promote the production of protective factors such as (Bcl-2, SOD2, G-CSF)

Based on the molecular mechanisms by which gut microbiota-derived products mediate radioprotection via multiple signaling pathways (Table 2), clinical interventions could establish a synergistic system of “microbiota remodeling-metabolic regulation”: on one hand, administering specific probiotics or performing FMT can restore the balance of the intestinal microecosystem; on the other hand, combining dietary interventions—such as a high-fiber diet to promote SCFAs production and tryptophan supplementation to enhance indole metabolite synthesis—ensures the continuous supply of endogenous protective substances. Through the synergistic effects of exogenous microbiota modulation and endogenous metabolic activation, this combined strategy may be more effective in maintaining intestinal homeostasis during radiotherapy, thereby forming a multi-level intestinal barrier protection. However, further clinical research is necessary to establish individualized intervention protocols (Eaton et al., 2022).

### 3.2 Gut microbiota and bioengineering enable radiosensitization

**Figure 2:**
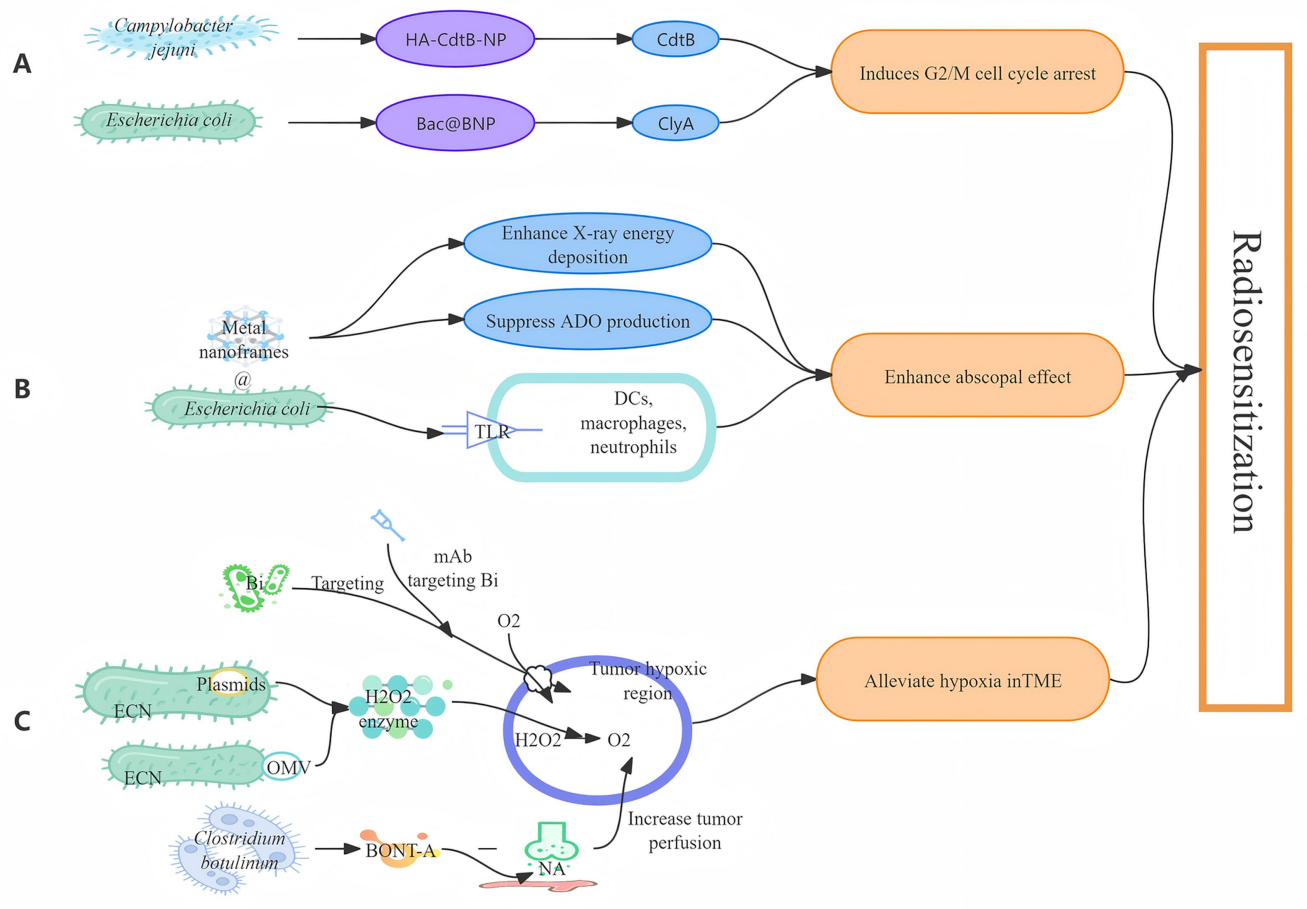
Radiosensitization strategies based on the gut microbiota and bioengineering. The integration of gut microbiota and bioengineering represents a future direction for enhancing radiosensitivity in radiotherapy, primarily involving three aspects: arresting the tumor cell cycle **(A)**; enhancing the abscopal effect **(B)**; improving the hypoxic tumor microenvironment **(C)**.

The development of tumor radioresistance involves multifaceted biological mechanisms, encompassing intrinsic tumor cell properties such as enhanced DNA repair, anti-apoptotic pathway activation, and metabolic reprogramming, as well as extrinsic factors within the Tumor Microenvironment (TME) including hypoxia and immunosuppression (Meads et al., 2009; Wu et al., 2023). Radiotherapy resistance not only leads to suboptimal treatment efficacy, worsened prognosis, and reduced quality of life but also compromises normal tissue function and increases the risk of secondary malignancies or long-term complications (Huang and Zhou, 2020). Although strategies combining chemotherapy, targeted therapy, or immunotherapy have yielded partial progress, clinical outcomes remain suboptimal due to limitations such as single-target effects, systemic toxicity, and acquired resistance (Kouhsari et al., 2018). Within this context, gut microbiota in conjunction with emerging nanomaterial and engineered bacterial technologies represent a novel research direction to overcome the bottleneck in radiosensitization (Figure 2).

Liu et al. demonstrated that tumor cells in the G2/M phase exhibit high radiosensitivity (Liu et al., 2019). Prolonging G2/M phase arrest represents an effective strategy for achieving radiosensitization. Gram-negative bacteria in the gut (e.g., *Campylobacter jejuni* and *Salmonella*) produce cytolethal distending toxin (CDT). Its CdtB subunit induces DNA double-strand breaks, specifically arresting the tumor cell cycle in the G2/M phase, thereby significantly enhancing tumor cell radiosensitivity (Lin et al., 2017). Chen’s team developed hyaluronic acid-modified nanoparticles encapsulating CdtB (HA-CdtB-NPs), which target the hyaluronic acid receptor CD44 on prostate cancer cells. This enables precise delivery of CdtB to prostate cancer cells, significantly improving radiotherapy efficacy (Chen Y. A. et al., 2021). Furthermore, cytolysin A (ClyA), a bacterial toxin secreted by Enterobacteriaceae (e.g., *Escherichia coli*), also induces G2/M phase cell cycle arrest (Wai et al., 2003). Building on this, Pan et al. engineered *Escherichia coli MG1655* to develop ClyA-overexpressing bacteria (Bac), which was co-assembled with synthetic nanoparticles (BNP) into a radiosensitizing nanosystem (Bac@BNP). Intravenous administration of Bac@BNP in breast cancer-bearing mice facilitated its accumulation at tumor sites and precise release of ClyA, markedly enhancing radiotherapy efficacy (Pan et al., 2022). Leveraging bacterial toxins derived from enteric pathogens, combined with bioengineering technologies, can transform them into effective strategies to overcome radioresistance.

While radiotherapy kills tumor cells, it can also trigger immunogenic cell death (ICD), releasing adenosine triphosphate (ATP). This activates anti-tumor immunity and generates an abscopal effect capable of killing tumors in non-irradiated areas (Wu et al., 2025). However, this process often leads to the accumulation of the immunosuppressive metabolite adenosine (ADO) (Zhu et al., 2025). Consequently, the anti-tumor immune response elicited by radiotherapy alone is limited. Combining radiotherapy with bacteria can effectively enhance this abscopal effect, thereby improving radiotherapeutic efficacy. Studies indicate that bacteria and their components can serve as PAMPs, recognized by TLR. This recognition activates immune cells such as dendritic cells, macrophages, and neutrophils, effectively enhancing the radiotherapy-induced abscopal effect (Huang X. et al., 2021). Nanomaterials and engineered bacteria derived from the gut microbiota play crucial roles in this strategy. Chen et al. synthesized a composite termed Au-OMVS using gold nanoparticles (Au NPs) and OMVS produced by *Escherichia coli*. Combined with radiotherapy, Au-OMVS significantly upregulated the expression of ROS and TNF-α, exerting both radiosensitizing and immunomodulatory effects (Chen M. H. et al., 2021). Wu et al. utilized *Escherichia coli Nissle 1917* (ECN) as a delivery vehicle, co-assembling it with a bismuth-based metal–organic framework (Bi-MOF) to construct a biohybrid system, EcN@Bi-MOF/POM-1. This system markedly promoted radiotherapy-induced ICD while limiting the degradation of ATP into immunosuppressive ADO, thereby enabling sustained antitumor immunity (Wu et al., 2025). Additionally, Deng et al. grafted nanoparticles (AZTF) onto probiotic surfaces, creating an engineered biohybrid, Bc@AZTF, which also effectively enhanced anti-tumor immunity (Deng et al., 2024). Collectively, these recent studies indicate that composites formed from nanomaterials and engineered gut microbiota-derived bacteria can significantly amplify the radiotherapy-induced abscopal effect, providing novel tools for achieving radiosensitization.

The hypoxic conditions prevalent within the TME constitute another critical barrier to radioresistance. As the specialized ecological system sustaining tumor cell survival, the hypoxic characteristics of the TME are closely associated with radioresistance (Arneth, 2019). Studies have shown that the hypoxic environment can increase the radiation resistance of tumor cells two- to three-fold by activating signaling pathways such as HIF-1 and VEGF (Wu et al., 2023). Exploiting the anaerobic properties of specific gut microbiota in conjunction with bioengineering technologies enables targeted overcoming of this critical radioresistance barrier. Leveraging the anaerobic nature of *Escherichia coli* and the facile genetic modifiability of its plasmid genes, Huang et al. engineered an ECN strain harboring a plasmid encoding catalase under the control of the strong tac promoter. When ECN accumulates within the hypoxic regions of tumors, it releases catalase, efficiently converting endogenous tumor H₂O₂ into O₂. This alleviates hypoxia in the TME and consequently enhances tumor radiosensitivity (Huang C. et al., 2021). Zai et al. developed catalase containing *E. coli* membrane vesicles (EMs). Delivered to hypoxic tumor regions, these EMs enhance catalase stability and catalytic efficiency by virtue of their nanostructure, generating substantial O₂ and significantly prolonging tumor oxygenation duration (Zai et al., 2021). Exploiting the natural hypoxic tropism of *Bifidobacterium* (Bi), Wu et al. developed a Bi-specific monoclonal antibody. By actively binding to Bi that accumulates in tumor hypoxic regions, this approach disrupts the hypoxic microenvironment while concurrently activating local immune responses, thereby inducing an anti-tumor abscopal effect (Wang W. et al., 2024; Yang et al., 2021). The disordered vascular system within tumor tissues is a primary cause of hypoxia (Phung et al., 2020), and enhancing blood perfusion in these tissues represents another strategy to ameliorate hypoxia. Ansiaux et al. found that the local injection of botulinum toxin A (BONT-A) within a human-safe dosage range effectively inhibits the release of noradrenaline from presynaptic membranes, leading to vasodilation and increased perfusion and oxygenation of hypoxic tumor regions, thereby creating a favorable microenvironment for conventional radiotherapy (Ansiaux et al., 2006). Therefore, leveraging the anaerobic properties of gut microbiota and their facile genetic modifiability, therapeutic agents can be targeted to hypoxic tumor regions to increase tumor perfusion. This alleviates hypoxia within the TME, ultimately achieving radiosensitization.

In summary, the integration of gut microbiota and biotechnology provides multidimensional strategies to overcome radioresistance (Table 3). These include leveraging bacterial toxins to induce tumor cell cycle arrest, enhancing immunogenic cell death and the abscopal effect via engineered bacteria and nanomaterials, and targeting the alleviation of hypoxia in the tumor microenvironment. Such interdisciplinary approaches enable precise modulation of tumor biology and the immune microenvironment, offering innovative and translationally promising solutions for radiosensitization.

**Table 3:** Mechanism of radiosensitization by gut microbiota and bioengineering.

References	Bacteria	Biohybrid	Study setting	Type of cancer	Mechanism
Chen Y. A. et al. (2021)	*Campylobacter jejuni*	HA-CdtB-NPs	*In vivo*	Prostate cancer	CdtB induces duplex DNA breaks that arrest the cell cycle in G2/M phase
Pan et al. (2022)	*Escherichia coli MG1655*	Bac@BNP	*In vivo*	Breast cancer	ClyA elevates the proportion of cells in both G2/M and G0/G1 phases concomitantly. High-Z elements further enhance X-ray energy deposition
Chen M. H. et al. (2021)	*Escherichia coli*	Au-OMVS	*In vitro*; *in vivo*	Glioblastoma	AU-OMVS enhances the effect of radiotherapy by promoting intracellular ROS generation, macrophage chemotaxis and activation, and TNF-α secretion in cancer cells, while gold nanoparticles also enhance X-ray energy deposition
Wu et al. (2025)	ECN	EcN@Bi-MOF/POM-1	*In vitro*; *in vivo*	Colorectal cancer; breast cancer	ECN, Bi-MOF enhance anti-tumor immunity; Bi enhances ray-damaging DNA capacity; POM-1 reduces immune cell suppression
Deng et al. (2024)	ECN	Bc@AZTF	*In vitro*; *in vivo*	Breast cancer	Leveraging the anaerobic tropism of ECN, AZTF nanoparticles were delivered to tumor sites to induce ICD while concurrently suppressing ADO production
Huang C. et al. (2021)	ECN	ECN	*In vitro*; *in vivo*	Breast Cancer	ECN delivers plasmids containing the tac strong promoter and H₂O₂ - degrading enzyme genes to the hypoxic regions of the tumor, catalyzing the production of O₂ from H₂O₂ in the TME to alleviate hypoxia
Zai et al. (2021)	ECN	EMs	*In vitro*; *in vivo*	Breast Cancer	Injecting EMs containing H2O2 enzymes into the tumor catalyzes the production of O_2_ from H_2_O_2_ generated by the tumor cells, thereby ameliorating hypoxia in TME
Wang W. et al. (2024)	*Bifidobacterium bifidum*	–	*In vitro*	Colorectal cancer; breast cancer	Injections of Bi conjugated mAb can enhance the efficacy of radiotherapy by colonizing and destroying hypoxic areas of the tumor
Ansiaux et al. (2006)	*Clostridium botulinum*	BONT-A	*In vitro*; *in vivo*	Fibrosarcoma; liver cancer	BONT-A elevates oxygenation in hypoxic areas by inhibiting norepinephrine release to dilate blood vessels

## 4 Summary and future perspectives

The bidirectional interaction between abdominopelvic radiotherapy and the gut microbiota is a critical nexus influencing both therapeutic efficacy and toxicities. Radiotherapy significantly alters the structure of the gut microbiota, and these alterations are closely associated with the occurrence of radiotherapy-related toxicities and the development of radioresistance. Gut microbiota, along with their metabolites and structural components, can activate various intestinal protective pathways. Leveraging their unique biological properties and combined with bioengineering techniques, the gut microbiota can also serve as an effective tool to overcome radioresistance. This interaction mechanism establishes the theoretical foundation for developing “microbiota-radiotherapy” synergistic strategies. Building upon this, microecological remodeling strategies—such as dietary modulation (e.g., increasing intake of high-fiber and tryptophan-rich foods), oral probiotics, and FMT—can promote the production of specific beneficial microbial derivatives. This provides clinically feasible interventions to mitigate radiotherapy-induced injury. However, further randomized controlled clinical trials are required to define the efficacy, safety, and individualized applicability criteria for probiotic, FMT, and metabolite-based interventions. Currently, novel strategies, including genetically engineered bacterial strains, metal nanomaterial-engineered bacteria composite systems, and nanocarrier-mediated targeted delivery of microbial metabolites, are emerging as cutting-edge directions in radiosensitization research. Future studies should focus on screening safe strains or developing attenuated engineered strains while addressing challenges such as the short half-life and genetic instability of engineered bacteria. Ultimately, gut microbiota modulation is poised to become a core component of optimized radiotherapy regimens. Through “microbiota-radiotherapy” synergistic strategies, it offers the potential to enhance efficacy while reducing toxicity, opening new dimensions in abdominopelvic cancer treatment.

## Glossary

ADO: Adenosine
AhR: Aryl hydrocarbon receptor
ATP: Adenosine triphosphate
Bi: Bifidobacterium
BONT-A: Botulinum toxin type A
CDT: Cytolethal distending toxin
ClyA: Cytolysin A
DIM: 3, 3′-diindolylmethane
ECN: *Escherichia coli Nissle 1917*
EMs: *E. coli* membrane vesicles
EPS-2: Exopolysaccharides-2
FMT: Fecal microbiota transplantation
HAT: Histone acetyltransferases
HDAC: Histone deacetylase
I3A: Indole-3-carboxaldehyde
ICD: Immunogenic cell death
IPA: Indole-3-propionic acid
KYNA: Kynurenic acid
LGG: *Lactobacillus rhamnosus GG*
LPS: Lipopolysaccharide
LTA: Lipoteichoic acid
MSCs: Mesenchymal stem cells
OMVs: Outer membrane vesicles
PAMPs: Pathogen-associated molecular patterns
RE: Radiation enteritis
ROS: Reactive oxygen species
SCFAs: Short-chain fatty acids
TLR: Toll-like receptor
TME: Tumor microenvironment
