# Correction: Interplay between the abdominopelvic radiotherapy and gut microbiota

**DOI:** 10.3389/fmicb.2025.1773904

**Published:** 2026-01-29

**Authors:** Ding Chenxiang, Zhuang Jing, Yu Xiaojian, Xie Hua, Yang Xi

**Affiliations:** 1 Huzhou Central Hospital, Fifth School of Clinical Medicine of Zhejiang Chinese Medical University, Huzhou, China; 2 Huzhou Central Hospital, Affiliated Central Hospital Huzhou University, Huzhou, China; 3 Bengbu Medical University Graduate School, Bengbu, China; 4 Xuancheng People's Hospital, Affiliated Xuancheng Hospital of Wannan Medical College, Xuancheng, China; 5 Zhejiang-France United Laboratory of Integrated Traditional Chinese and Modern Medicine in Colorectal Cancer, Huzhou, China

**Keywords:** gut microbiota, radiotherapy, abdominopelvic malignant tumor, radiation-induced intestinal injury, radiosensitization

In the published article, the following affiliation errors were identified:

Affiliation 1 “Huzhou Central Hospital, Fifth School of Clinical Medicine of Zhejiang Chinese Medical University” was omitted for authors Zhuang Jing, Yu Xiaojian, and Yang Xi.

Affiliation 2, “Huzhou Central Hospital, Affiliated Central Hospital Huzhou University” was omitted for author Ding Chenxiang.

Affiliation 3, “Bengbu Medical University Graduate School” was omitted for author Ding Chenxiang. Affiliation 3 was erroneously assigned to authors Zhuang Jing, Yu Xiaojian and Yang Xi.

Affiliation 4, “Xuancheng People's Hospital, Affiliated Xuancheng Hospital of Wannan Medical College” was omitted for authors Ding Chenxiang and Xie Hua. Affiliation 4 was erroneously assigned to authors Zhuang Jing, Yu Xiaojian and Yang Xi.

Affiliation 5, “Zhejiang-France United Laboratory of Integrated Traditional Chinese and Modern Medicine in Colorectal Cancer” was omitted for authors Zhuang Jing, Yu Xiaojian, and Yang Xi. Affiliation 5 was erroneously assigned to author Xie Hua.

The original version of the article has been updated.

